# Hypoplastic Vertebral Artery Contributes to Stroke by Promoting Progression of Basilar Artery Bending

**DOI:** 10.1002/brb3.71488

**Published:** 2026-05-14

**Authors:** Jie Li, Xiaogang He, Hui Liu, Zeming Ye, Guanfeng Zeng, Peiqun Yang, Juehua Zhu, Zheng Dai, Qin Fu, Yongjun Jiang

**Affiliations:** ^1^ Department of Neurology Wuxi People's Hospital Wuxi Medical Center The Affiliated Wuxi People's Hospital of Nanjing Medical University Nanjing Medical University Wuxi China; ^2^ Department of Neurology The Second Affiliated Hospital Guangzhou Medical University Guangzhou China; ^3^ Department of Neurology Kunshan Hospital of Traditional Chinese Medicine Kunshan China; ^4^ Department of Neurology Changshu No.2 People's Hospital, Affiliated Changshu Hospital of Nantong University Changshu China; ^5^ Department of Neurology Guangzhou Panyu District Hexian Memorial Hospital Guangzhou China; ^6^ Department of Neurology Guangzhou Hospital of Integrated Traditional and Western Medicine Guangzhou China; ^7^ Department of Neurology The First Affiliated Hospital of Soochow University Suzhou China

**Keywords:** basilar artery bending, hypoplastic vertebral artery, POCI

## Abstract

**Background:**

As a well‐recognized vascular variation, hypoplastic vertebral artery has been recognized as a potential risk for stroke. We aimed to assess the odds of new‐onset stroke over a 20‐year period in subjects with versus without hypoplastic vertebral artery, exploring how the progression of basilar artery bending mediates the odds.

**Methods:**

This was a retrospective cohort study, with 20 years of follow‐up (2004–2024, analysis performed in June 2025). The primary outcome was the occurrence of new‐onset posterior circulation infarction during follow‐up. Logistic regression models were fitted for new‐onset posterior circulation infarction as gender, baseline stroke, baseline posterior circulation infarction, basilar artery stenosis during follow‐up, the progression of basilar artery bending, and hypoplastic vertebral artery. Mediation analyses were conducted for vertebral artery stenosis during follow‐up, basilar artery stenosis during follow‐up, and the progression of basilar artery bending.

**Results:**

A total of 1464 subjects were included, 547 with hypoplastic vertebral artery. During the 20‐year follow‐up, 91 participants had new‐onset posterior circulation infarction. The occurrence of new‐onset posterior circulation infarction was significantly associated with progression of basilar artery bending (odds ratio 1.700, 95% confidence interval 1.032–2.798, *p *= 0.037) and hypoplastic vertebral artery (odds ratio 1.481, 95% confidence interval 1.019–2.003, *p *= 0.015). The proportion of new‐onset posterior circulation infarction attributable to hypoplastic vertebral artery was mediated by the progression of basilar artery bending, accounting for approximately 50.2% (95% confidence interval 3.2%–102.4%).

**Conclusions:**

Results of this study revealed that hypoplastic vertebral artery was associated with new‐onset posterior circulation infarction, and progression of basilar artery bending may serve as a potential intermediate factor in this association.

## Graphical abstract

Progression of basilar artery bending and hypoplastic vertebral artery were significantly associated with stroke onset.

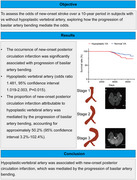

## 1 Introduction

Approximately 40% of the population exhibits asymmetrical diameters in their two vertebral arteries (VAs) (X. Chen et al. 2021). If the side‐to‐side diameter difference‐value exceeds 0.3 mm, the smaller one is defined as a hypoplastic VA, or when the diameters both VAs are equal, the dominant VA is identified as the side that connects more closely with the basilar artery (BA) (Hong et al. 2009). Historically, hypoplastic VA was regarded as a normal congenital vascular anomaly. However, increasing evidence has demonstrated that hypoplastic VA constitutes a significant risk factor for stroke (Sun et al. 2022, W. Zhu et al. 2016). W. Zhu et al. found that patients with hypoplastic VA had a 2‐fold increased risk of stroke compared to those without hypoplastic VA (W. Zhu et al. 2016). The underlying mechanism of hypoplastic VA for stroke remains unclear.

BA arises from the union of the bilateral VAs (Wu et al. 2024, J. Zhu et al. 2022). The blood flow in the BA is streamlined when the bilateral VAs are symmetrical. In patients with hypoplastic VA, the flow rate from asymmetric VAs becomes unequal, resulting in turbulent blood flow within the BA (Huang et al. 2022). The turbulent flow may lead to morphological deformation. Hypoplastic VA has been related to BA bending, with the direction of bending corresponding to that of hypoplastic VA (Hong et al. 2009). Nevertheless, these conclusions are based on cross‐sectional studies, and no longitudinal cohort studies have been conducted to validate these findings. The impact of hemodynamic changes on morphological deformation appears to be minimal and gradual, necessitating an extended observation period. BA bending has been related to stroke occurrence (Hong et al. 2009, Ngo et al. 2020), typically manifesting as an infarction located contralateral to the direction of BA bending. Whether hypoplastic VA induces stroke through mechanisms related to BA bending remains undetermined.

In the present study, we aimed to investigate whether hypoplastic VA was a risk factor for stroke onset during a long‐term follow‐up, mediated by the progression of BA bending.

## 2 Methods

### 2.1 Study Population

The research protocol of this retrospective, hospital‐based, longitudinal cohort study was reviewed and approved by the ethics committees of each center (the Second Affiliated Hospital of Guangzhou Medical University 2015‐WZ‐23, Changshu No.2 People's Hospital 2024‐013, the First Affiliated Hospital of Soochow University 201600015, and Wuxi People's Hospital LCSY‐2016–04‐003) according to the principles expressed in the Declaration of Helsinki. Written informed consent was obtained from patients or their authorized representatives before enrollment.

### 2.2 Experimental Design

Patients with neurological symptoms or signs admitted to the centers from June 1, 2004, to December 31, 2024, were consecutively screened. All subjects underwent standard magnetic resonance imaging (MRI) scan, including T1‐weighted imaging, T2‐weighted imaging, diffusion‐weighted imaging (DWI), fluid‐attenuated inversion recovery sequence, and apparent diffusion coefficient maps. Magnetic resonance angiography (MRA) was applied to evaluate intracranial and extracranial arteries.

Patients were enrolled if they met the inclusion criteria as follows: (1) older than 18 years; (2) the interval between the first and last MRI scanning should be at least 10 years (3600 days); (3) no significant stenosis in the VAs or BA. Patients were excluded if they met any of the following criteria: (1) no following neuroimages; (2) the whole VAs was not covered in the index and following neuroimages; (3) missing clinical or imaging data, or poor quality of neuroimages; (3) isolated ICVA.

### 2.3 Data Collection

Trained interviewers conducted face‐to‐face interviews at each center to collect data at baseline, which consisted of demographic information (including age and gender), risk factors for stroke (including smoking, drinking, body mass index [BMI], hypertension, diabetes, previous stroke or transient ischemic attack [TIA], coronary heart disease [CHD] history, atrial fibrillization, and tumor history), and medications (including antihypertensive agents, hypoglycemic agents, lipid‐lowering agents, antiplatelet agents, and anticoagulant agents). Laboratory examinations (including total cholesterol, TG, high‐density lipoprotein [HDL] cholesterol, and low‐density lipoprotein [LDL] cholesterol) were performed after 12 h of overnight fasting.

The arteries were evaluated by 3D time‐of‐flight MRA (3D TOF‐MRA) at baseline and follow‐up. Patients were scanned with a 1.5 Tesla (T) or 3.0T MRI unit (Philips or Siemens). We used 3D TOF‐MRA acquired with a 3D transversal fast low angle shot sequence with flow compensation; repetition time/echo time 15/3.5 ms for 3T MRI (24/7.2 ms for 1.5T), parallel imaging acceleration Factor 3 for 3T MRI (2 for 1.5T MRI), field of view 220 × 180 mm for 3T MRI (240 × 200 mm for 1.5T MRI), slice thickness 1.2 mm, six slabs with 24 slices each and reconstructed image resolution of 0.3 × 0.3 × 0.5 mm for 3T MRI (slice thickness 0.55 mm, four slabs with 40 slices each and reconstructed image resolution of 0.4 × 0.4 × 0.6 mm for 1.5T MRI). Volume‐rendered reconstructions and maximum intensity projection reconstructions of source images were used to optimize stenosis detection. Reconstructions were conducted, and images were evaluated blindly by two senior neuroradiologists (HL and ZD). In cases of inter‐evaluator disagreement, a third neuroradiologist in the core lab (the Second Affiliated Hospital of Guangzhou Medical University) arbitrated the discrepancies.

### 2.4 Variables

Drinking was defined as heavy intake (≥ 14 drinks/week in women or 21 drinks/week in men) or episodic heavy intake (≥ 5 drinks/episode at least once per month). Hypertension was defined as SBP ≥ 140 mmHg or DBP ≥ 90 mmHg, any use of antihypertensive drugs, or a self‐reported history of hypertension. Diabetes mellitus was defined as HbA1c ≥ 6.5%, or FBG levels ≥ 7.0 mmol/L, or blood glucose ≥ 11.1 mmol/L at 2‐h oral glucose tolerance test, or blood glucose ≥ 11.1 mmol/L at random glucose test with symptoms of diabetes, or any use of hypoglycemic agents, or any self‐reported history of diabetes.

The infarction locations were classified according to the Oxfordshire Community Stroke Project Classification (Kopyta et al. 2020), and included the following: total anterior circulation infarct (TACI), partial anterior circulation infarct (PACI), and posterior circulation infarct (POCI). TACI is a type of large cerebral infarction affecting the entire anterior circulation supplied by the internal carotid artery. PACI is a less severe stroke that affects only part of the anterior circulation, and POCI describes any infarct in an area of the brain supplied by the posterior circulation. Considering the relatively low rate of BA bending progression, recurrent stroke was defined as any new infarction on MRI identified at least 3000 days after index MRI scanning. If multiple instances of new‐onset stroke occurred, the first time was recorded and used for analysis.

The degree of BA bending was evaluated based on the lateral‐most position of the BA throughout its course as follows: 0, midline; 1, medial to lateral margin of the clivus or dorsum sellae (1.02–2.68 mm); 2, lateral to the lateral margin of the clivus or dorsum sellae (2.69–3.76 mm); and 3, in the cerebellopontine angle cistern (≥ 3.77 mm) (Smoker et al. 1986). BA bending progression was defined as an elevation from a lower grade at baseline to a higher grade at follow‐up, such as from Grade 1 to Grade 3. Stenosis of ICVA or BA was defined as significant stenosis (≥ 50%) using the WASID method (Chimowitz et al. 2005). Hypoplastic VA was defined as the intracranial segment of the VA that is smoothly narrowed, with a diameter less than half that of the contralateral side (side‐to‐side diameter difference ≥ 0.3 mm), or an absolute diameter < 2 mm (Liu et al. 2020). In our cohort, all VAH cases were unilateral, as bilateral VAH is rare and was not identified in our sample. Isolated VA was defined as VA not communicating with the BA but ending into the ipsilateral PICA according to MRA and verified by DSA (J. Zhu et al. 2022).

### 2.5 Statistical Analyses

Statistical analysis was performed using SPSS (version 20.0). A two‐sided *p* < 0.05 was considered statistically significant. Continuous variables were tested for normality using the Kolmogorov–Smirnov test. Normally distributed continuous variables were described as the mean ± standard deviation, while categorical variables were expressed as percentages. Group differences were analyzed using the Kruskal–Wallis test for continuous variables, and the chi‐square test for categorical variables. Logistic regression was used to analyze the association between hypoplastic VA and recurrent POCI, by calculating odds ratio (OR) and 95% confidence interval (CI). Kaplan–Meier event‐free survival curves were generated, and the significance was evaluated by log‐rank tests.

**FIGURE 1:**
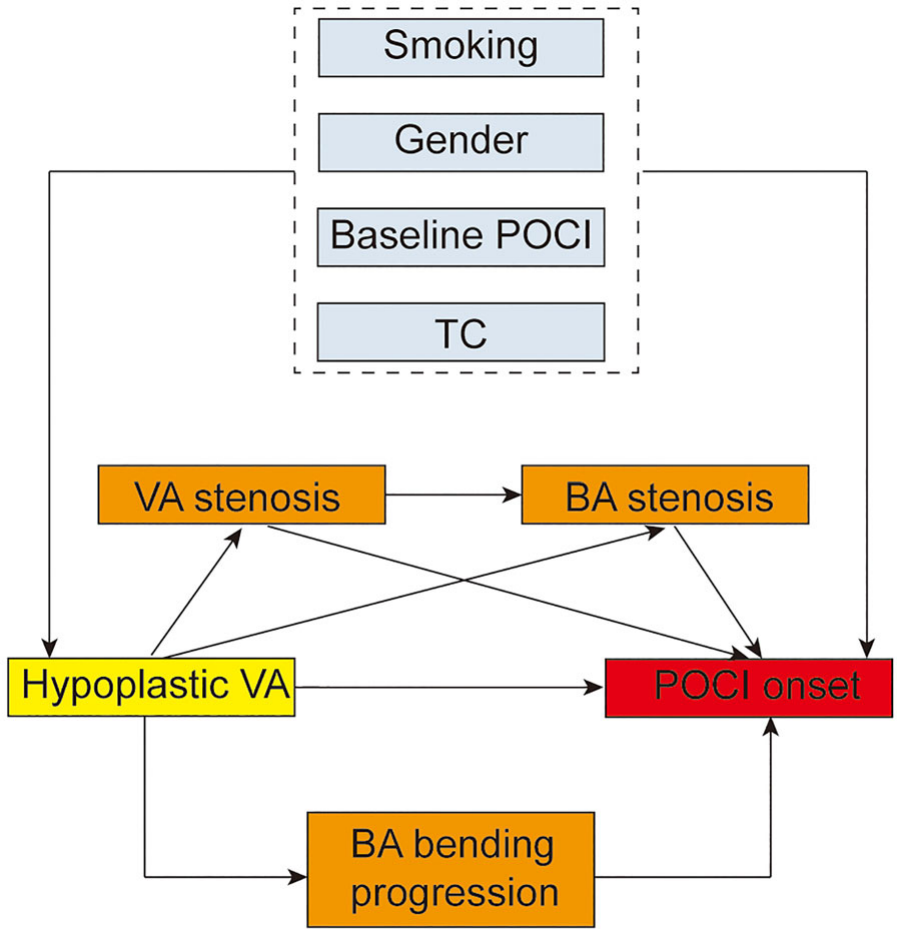
Simplified directed acyclic graph of hypothesized causal mechanisms from hypoplastic VA to new‐onset POCI. Indirect effects are derived from product of coefficients in two regression models (mediator ∼ exposure; outcome ∼ exposure + mediator). Structural equation modeling quantifies direct and indirect paths among hypoplastic VA, VA stenosis, BA stenosis, BA bending progression, and recurrent POCI. Proportion mediated (95% CI) is shown for VA stenosis, BA stenosis, and BA bending progression.

From the DAG (Figure 1), several mediators were identified, for which medication analyses were performed using the mediation R package (Li et al. 2024). In brief, the indirect effect was obtained by multiplying the coefficients of two regression models: one calculating the mediator on the exposure and the other for the outcome on both the exposure and the mediator. Structural equation modeling was used to measure direct and indirect relationships between variables and to ascertain the structure among hypoplastic VA, VA stenosis, BA stenosis, BA bending progression, and recurrent POCI. The proportion mediated (95% CI) was calculated for three mediators: VA stenosis, BA stenosis, and BA bending progression.

## 3 Results

**FIGURE 2:**
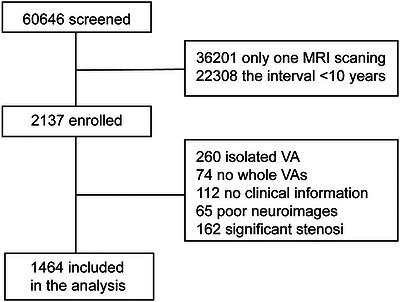
Flow chart of patient recruitment.

As shown in Figure 2, 1464 subjects (mean age, 63.14 ± 10.40 years; 714 male [48.8%]) were finally included, 547 with hypoplastic VA (37.4%). The median follow‐up was 10.92 ± 2.23 years.

### 3.1 Descriptive Analyses

A general description of the subjects is listed in Table 1. Males and smokers were more likely to have hypoplastic VA. Although no significant difference in the overall incidence of stroke was observed between the two groups, patients with hypoplastic VA exhibited a higher prevalence of POCI (10.45% vs. 7.1%, *p* < 0.05, Table 1). BA bending was more commonly observed in patients with hypoplastic VA; furthermore, a greater number of patients with hypoplastic VA experienced progression of BA bending (28.2% vs. 13.0%, *p *< 0.05, Table 1). Interestingly, no significant differences in VA stenosis or BA stenosis during follow‐up were observed between patients with and those without hypoplastic VA. A higher incidence of new‐onset POCI was observed during follow‐up among patients with hypoplastic VA (8.2% vs. 5.0%, *p* < 0.05, Table 1).

**TABLE 1:** Baseline characteristics.

Characteristic	Normal VA	Hypoplastic VA	*p*	95% CI
No of subjects	917	547		
Age (y)	63.07 ± 10.54	63.27 ± 10.15	0.720	−1.304–0.900
Male	425 (46.3%)	289 (52.8%)	0.016	1.049–1.603
Risk factors				
BMI (kg/m^2^)	24.09 ± 3.72	24.17 ± 3.56	0.746	−0.554–0.397
Smoking	151 (16.5%)	113 (20.7%)	0.044	1.008–1.732
Drinking	59 (6.4%)	30 (5.5%)	0.462	0.537–1.327
Hypertension	522 (56.9%)	313 (57.2%)	0.912	0.817–1.254
Diabetes	178 (19.4%)	110 (20.1%)	0.745	0.801–1.363
Hyperlipidemia				
TC (mM)	4.64 ± 1.09	4.79 ± 1.19	0.036	−0.290—0.010
TG (mM)	1.56 ± 1.08	1.69 ± 2.29	0.234	−0.324–0.079
HDL‐c (mM)	1.09 ± 0.28	1.09 ± 0.27	0.903	−0.033–0.037
LDL‐c (mM)	2.97 ± 0.87	3.05 ± 0.92	0.150	−0.192–0.029
Medication				
Antihypertensive agents	433 (47.2%)	259 (47.3%)	0.962	0.813–1.243
Hypoglycemic agents	172 (18.8%)	102 (18.6%)	0.958	0.757–1.303
Lipid‐lowering agents	310 (33.8%)	187 (34.2%)	0.882	0.813–1.272
Antiplatelet agents	349 (38.1%)	202 (36.9%)	0.666	0.766–1.186
Anticoagulant agents	16 (1.7%)	11 (2.0%)	0.714	0.532–2.509
Medical History				
Stroke or TIA	368 (40.1%)	210 (28.4%)	0.510	0.748–1.155
CHD history	107 (11.7%)	51 (9.3%)	0.162	0.548–1.106
Atrial fibrillation	12 (1.3%)	9 (1.6%)	0.600	0.528–3.014
Tumor	79 (8.6%)	38 (6.9%)	0.255	0.530–1.184
New‐onset stroke	236 (25.7%)	163 (29.8%)	0.091	0.968–1.550
POCI	65 (7.1%)	57 (10.4%)	0.026	1.050–2.213
Baseline BA bending	123 (13.4%)	195 (36.6%)	< 0.001	
Grade 1	86 (9.4%)	123 (22.5%)		
Grade 2	26 (2.8%)	50 (9.1%)		
Grade 3	11 (1.2%)	22 (4.0%)		
VA stenosis follow‐up	144 (15.7%)	71 (13.0%)	0.154	0.589–1.088
BA stenosis follow‐up	188 (20.5%)	124 (22.7%)	0.332	0.878–1.467
BA bending progression	119 (13.0%)	154 (28.2%)	< 0.001	2.010–3.435
New‐onset stroke	127 (13.8%)	86 (15.7%)	0.326	0.862–1.561
New‐onset POCI	46 (5.0%)	45 (8.2%)	0.014	0.109–2.597
Medulla	2 (0.2%)	3 (0.5%)		
Pons	26 (2.8%)	17 (3.1%)		
Midbrain	4 (0.4%)	3 (0.5)		
Cerebellum	6 (0.6%)	7 (1.3%)		
Occipital lobe and thalamus	8 (0.9%)	15 (2.7%)		

*Note*: *p *< 0.05 was considered statistically significant. Continuous variables were tested for normality using the Kolmogorov–Smirnov test. Normally distributed continuous variables were described as the mean ± standard deviation, while categorical variables were expressed as percentages. Group differences were analyzed using the Kruskal–Wallis test for continuous variables, and the chi‐square test for categorical variables.

Abbreviations: BA, basilar artery; BMI, body mass index; CHD, coronary artery disease; HDL‐c, high‐density lipoprotein cholesterol; LDL‐c, low‐density lipoprotein cholesterol; POCI, posterior circulation infarction; TC, total cholesterol; TG, triglyceride; TIA, transient ischemic attack; VA, vertebral artery.

### 3.2 Multiple Regression Analyses

Table 2 summarizes the results of the univariable and multivariable analyses. Over 20 years, a total of 213 patients developed a new‐onset stroke, with 91 in the posterior circulation area. The ORs of new‐onset POCI were significantly higher in patients with versus without acute stroke at baseline (OR 1.974, 95% CI 1.174–3.322, *p* = 0.010, Table 2), BA stenosis during follow‐up (OR 2.295, 95% CI 1.459–3.609, *p* < 0.001, Table 2), BA bending progression (OR 1.700, 95% CI 1.032–2.798, *p* = .037, Table 2), and hypoplastic VA (OR 1.481, 95% CI 1.019–2.003, *p* = 0.015, Table 2) when adjusting for gender and basline POCI.

**TABLE 2:** Comparison of baseline characteristics between patients with and without POCI recurrence.

	No recurrence	Recurrence	Univariate analysis			Multivariate analysis		
	*N* = 1373	*N* = 91	OR	*p*	95% CI	OR	*p*	95% CI
Age	63.15 ± 10.36	63.06 ± 11.00	0.999	0.936	0.979–1.02			
Male (y)	655 (47.7%)	59 (64.8%)	2.021	0.002	1.298–3.148	1.548	0.066	0.971–2.466
Risk factors								
BMI (kg/m^2^)	24.11 ± 3.69	24.40 ± 2.85	1.021	0.599	0.944–1.106			
Smoking	242 (17.6%)	22 (24.2%)	1.490	0.118	0.904–2.456			
Drinking	82 (6.0%)	7 (7.7%)	1.312	0.507	0.588–2.928			
Hypertension	787 (57.3%)	48 (52.7%)	0.831	0.394	0.543–1.272			
Diabetes	267 (19.4%)	21 (23.1%)	1.423	0.400	0.749–2.060			
Hyperlipemia								
TC (mM)	4.70 ± 1.14	4.60 ± 1.08	0.925	0.526	0.727–1.177			
TG (mM)	1.60 ± 1.64	1.78 ± 1.38	1.048	0.429	0.933–1.178			
HDL‐c (mM)	1.10 ± 0.28	1.05 ± 0.27	0.561	0.266	0.203–1.553			
LDL‐c (mM)	3.00 ± 0.89	2.94 ± 0.88	0.927	0.623	0.684–1.255			
Medication								
Antihypertensive agents	654 (47.6%)	38 (41.8%)	0.788	0.278	0.513–1.212			
Hypoglycemic agents	253 (18.4%)	21 (23.1%)	1.328	0.272	0.800–2.204			
Lipid‐lowering agents	461 (33.6%)	36 (39.6%)	1.295	0.244	0.838–2.000			
Antiplatelet agents	510 (37.1%)	41 (45.0%)	1.388	0.133	0.905–2.127			
Anticoagulant agents	27 (2.0%)	0 (0%)	< 0.001	0.998	—			
Medication history								
Stroke or TIA	545 (39.7%)	33 (36.3%)	0.864	0.517	0.556–1.343			
CHD history	149 (10.8%)	9 (9.9%)	0.902	0.775	0.444–1.832			
Atrial fibrillation	21 (1.5%)	0 (0%)	< 0.001	0.998	—			
Tumor	108 (7.9%)	9 (9.9%)	1.286	0.492	0.628–2.630			
New‐onset stroke	352 (25.6%)	47 (51.6%)	3.098	< 0.001	2.018–4.756	1.974	0.010	1.174–3.322
POCI	102 (7.4%)	20 (22.0%)	3.510	< 0.001	2.055–5.997	1.729	0.091	0.916–3.263
Baseline BA bending			1.210	0.179	0.916–1.598			
Grade 1	193 (14.0%)	16 (17.6%)						
Grade 2	70 (5.1%)	6 (6.6%)						
Grade 3	30 (2.2%)	3 (3.3%)						
VA stenosis follow‐up	198 (14.4%)	17 (18.7%)	1.363	0.268	0.788–2.359			
BA stenosis follow‐up	275 (20.0%)	37 (40.6%)	2.733	< 0.001	1.763–4.238	2.295	< 0.001	1.459–3.609
BA bending progression	247 (18.0%)	26 (28.6%)	1.823	0.013	1.134–2.933	1.700	0.037	1.032–2.798
Hypoplastic VA	502 (36.6%)	45 (49.4%)	1.545	0.015	1.019–2.365	1.481	0.027	1.019–2.003

*Note*: *p* < 0.05 was considered statistically significant. Logistic regression was used to analyze the association of hypoplastic VA and recurrent POCI, by calculating the odds ratio (OR) and 95% confidence interval (CI). The significant differences identified in the univariate analysis were included in the multivariate analysis.

Abbreviations: BA, basilar artery; BMI, body mass index; CHD, coronary artery disease; HDL‐c, high‐density lipoprotein cholesterol; LDL‐c, low‐density lipoprotein cholesterol; POCI, posterior circulation infarction; TC, total cholesterol; TG, triglyceride; TIA, transient ischemic attack; VA, vertebral artery.

**FIGURE 3:**
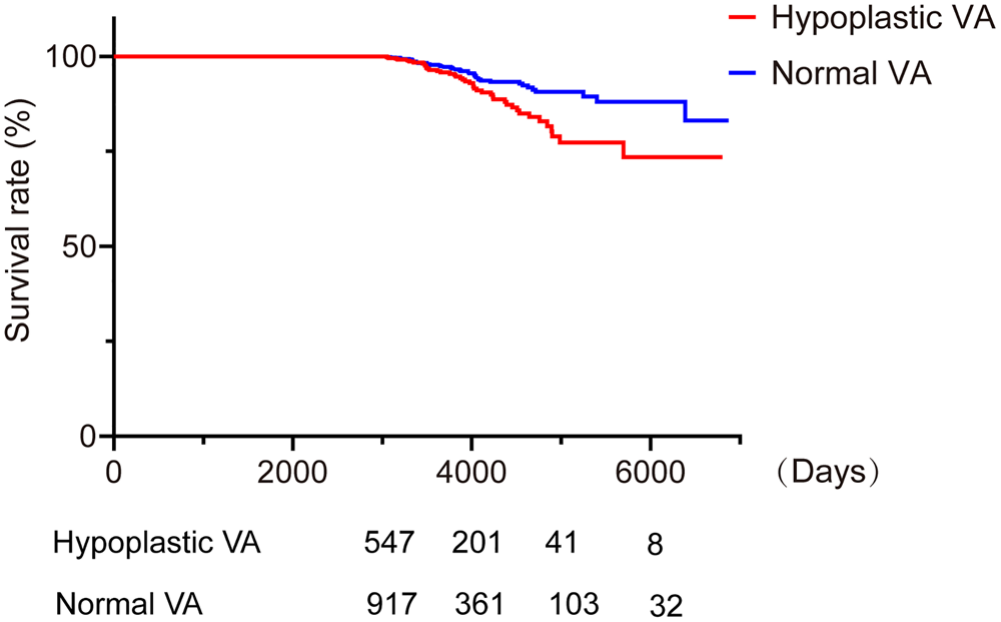
Kaplan–Meier event‐free survival curves for the hypoplastic VA group and the normal group, with significance evaluated using the log‐rank test.

The Kaplan–Meier survival (POCI‐free) curves by hypoplastic VA also showed statistically significant differences (log‐rank test, *p *= 0.003, Figure 3). Patients without hypoplastic VA presented higher event‐free survival (Figure 3).

### 3.3 Mediation Analyses

Table 3 presents the results of the mediation analyses presuming no exposure‐mediator interactions. The proportion of the effect of hypoplastic VA mediated by BA bending progression was 50.2% (95% CI 3.2%–102.4%) when adjusting for gender, acute stroke, and POCI at baseline, as well as TC. The VA stenosis and BA stenosis did not show significant mediation effects.

**TABLE 3:** Proportions mediated by different variables of hypoplastic VA with recurrent POCI.

Variable	Proportion mediated (%)	95% CI
VA stenosis	96.0	−13.6–213.6
BA stenosis	12.2	−12.5–40.4
BA bending progression	50.2	3.2–102.4

Abbreviations: BA, basilar artery; POCI, posterior circulation infarction; VA, vertebral artery.

## 4 Discussion

In the present study, we found that hypoplastic VA, BA stenosis, and BA bending progression were risk factors for new‐onset POCI at a median follow‐up of 10.9‐years; hypoplastic VA caused the new‐onset POCI mediated by BA bending progression but not BA stenosis. The strength of our study was that it demonstrated the role of hypoplastic VA in stroke onset and illustrated the possible mechanism in a longitudinal cohort study with a substantial sample size.

Hypoplastic VA was frequently observed in patients with stroke as well as in healthy individuals. In our cohort, one‐third of patients exhibiting neurological symptoms or signs were found to have hypoplastic VA, a finding that aligns with previous research. A hospital‐based study indicated that all patients admitted to the emergency department underwent MRI scanning, revealing that approximately one‐quarter of these individuals presented with hypoplastic VA (Freund et al. 2008). Patients with anomalous aortic origins or abnormal entrances at the transverse foramen are particularly predisposed to having hypoplastic VA (Kim et al. 2017). Furthermore, evidence suggests potential X‐linked inheritance patterns associated with hypoplastic VA based on a study involving 36 parent‐offspring pairs (Demarin et al. 2001). In patients with stroke, especially POCI, the prevalence of hypoplastic VA was significantly h higher. Among 112 POCI patients, 58 (51.8%) exhibited hypoplastic VA, including bilateral high‐variability arteries in 10 cases (Park et al. 2007). Our data also demonstrated an association between hypoplastic VA and POCI at baseline. Over 20 years of follow‐up, 23.9% of patients with hypoplastic VA experienced new‐onset strokes, among which 34.3% occurred within the posterior circulation area. Therefore, our data indicated that hypoplastic VA was a risk for stroke onset.

The mechanisms underlying stroke onset in patients with hypoplastic VA remain unknown. Considering the established role of atherosclerosis in stroke, we initially investigated the impact of hypoplastic VA on vertebrobasilar artery stenosis. However, our data showed no significant difference in VA stenosis during follow‐up. While BA stenosis was found to be predictive of stroke occurrence, there was no observed correlation between BA stenosis and hypoplastic VA. Our data were supported by others, which showed that the BA diameter of patients with hypoplastic VA showed no significant difference from that of those with normal VAs (Feng et al. 2020). In a cross‐sectional study, Park et al. found that hypoplastic VA was associated with a tendency toward multiple and extensive lesions, as well as an increased incidence of steno‐occlusion on the contralateral side of the hypoplastic VA (Park et al. 2007). This discrepancy may be attributed to variations in cohort characteristics and sample sizes across studies.

Previous studies have found a close relationship between hypoplastic VA and BA bending (Sahin and Gokce 2023). BA bending has been identified as an independent predictor of pontine infarction (Zhang et al. 2014). When BA bending was coupled with other vascular risk factors, the likelihood of pontine infarction increased (Zhang et al. 2014). In our study, it was found that hypoplastic VA was associated with BA bending. Furthermore, the degree of BA bending developed more in patients with hypoplastic VA and the BA bending progression was an independent predictor for POCI. As for the BA bending direction, there was an adverse directional correlation between the side of the dominant VA and BA bending (Sahin and Gokce 2023, Zheng et al. 2021). The underlying mechanism for BA bending was attributed to the significantly reduced dynamic blood flow volume in the hypoplastic VA, while the contralateral VA exhibited increased compensatory flow volume (Y. Y. Chen et al. 2010); additionally, hypoplastic VA demonstrated lower CO_2_ reactivity (Sato et al. 2015). In the present study, we used mediation analyses to identify that hypoplastic VA induced stroke onset by facilitating BA bending progression.

### 4.1 Limitations

First, this study was based on MRI findings. Patients who were unable to undergo MRI scanning were excluded, which may have introduced selection bias. Second, it was a hospital‐based study involving patients who received MRI scanning due to the presence of neurological symptoms or signs. Consequently, the incidence and clinical significance of hypoplastic VA in really normal people remains undetermined. Third, stroke in the present study was defined based on the many patients who presented with DWI‐negative strokes. The rationale for employing strict criteria was that symptoms associated with posterior circulation issues—such as dizziness or isolated vertigo—were common; however, these symptoms are rarely utilized for stroke diagnosis when DWI findings are negative. Moreover, we did not systematically classify recurrent strokes into etiological subtypes (e.g., cardioembolic, large artery, lacunar, or undetermined) because routine clinical workup for each recurrent event was not uniformly performed across all centers over the 20‐year follow‐up. Fourth, the role of collateral circulation, particularly posterior communicating arteries (Pcom) and fetal‐type posterior cerebral artery (PCA) was missing because routine MRA protocols did not consistently provide sufficient spatial resolution for reliable Pcom or fetal‐type PCA evaluation. Future prospective studies should incorporate high‐resolution MRA or CTA to evaluate the influence of collaterals on the relationship between VAH, BA bending, and stroke risk. Fifth, this was a retrospective longitudinal study based on routine MRA, and flow‐related sequences were not available. Future studies incorporating phase‐contrast MRI or 4D flow MRI to directly test the hemodynamic hypothesis are warranted. Sixth, we did not perform the animal model to investigate the effect of hypoplastic VA on BA bending and stroke onset. Finally, although our mediation analysis suggested that BA bending progression might account for approximately 50.2% of the association between hypoplastic VA and new‐onset POCI, the wide 95% CI (3.2%–102.4%) indicated considerable statistical instability. This imprecision likely arises from the relatively small number of POCI events (*n* = 91), the long follow‐up period, and the heterogeneous nature of posterior circulation strokes. Moreover, the lack of systematic etiological subtyping (e.g., cardioembolic, large artery, and lacunar) violates some assumptions of the mediation model. Therefore, we do not claim a robust mediated proportion; rather, we conclude that BA bending progression may serve as a *potential intermediate factor* that warrants further investigation in prospective studies with larger sample sizes and more precise hemodynamic assessments (e.g., 4D flow MRI).

## 5 Conclusions

Results of this study revealed that hypoplastic VA was associated with new‐onset POCI. Progression of BA bending may represent a potential intermediate factor in this association, but the mediation effect was imprecise and should be interpreted cautiously. Future studies are needed to validate this mechanistic pathway.

## Author Contributions

**Jie Li**: data curation. **Zeming Ye**: data curation. **Hui Liu**: data curation. **Zheng Dai**: methodology, writing – review and editing. **Xiaogang He**: data curation. **Qin Fu**: writing – review and editing. **Guanfeng Zeng**: data curation. **Yongjun Jiang**: writing – review and editing, conceptualization, methodology, supervision, formal analysis. **Juehua Zhu**: data curation. **Peiqun Yang**: data curation. All authors have read and approved the submitted version of the manuscript, and agreed to be personally accountable for their own contributions and questions related to the accuracy or integrity of any part of the work.

## Funding

This study was financially supported by the National Natural Science Foundation of China (82371345 and 82571487), Guangdong Basic and Applied Basic Research Foundation (2024A1515013188), Guangzhou Science and Technology Project (2024A03J0941), and Grant for clinical trial and cohort study of Wuxi Medical Center of Nanjing Medical University (WMCC202506) to Yongjun Jiang. The funding sources had no involvement in the design of the study, in the collection, analysis, interpretation of data, or in writing the manuscript.

## Conflicts of Interest

The authors declare no conflicts of interest.

## Data Availability

Data is accessible to authorized users and applications when and where they need it.
